# Supplementary material for: Physiological Mechanism through Which Al Toxicity Inhibits Peanut Root Growth
Source: Plants (Basel). 2024 Jan 22;13(2):325. doi: 10.3390/plants13020325 (PMC10820445; doi:10.3390/plants13020325)
Supplement: Supplementary file 1 [file plants-13-00325-s001.zip › Table S1-S3.pdf]

Table S1. Gradient parameters of HPLC

Time (min)	Flow velocity (mL/min)	A%
0-1	0.3	20
1-3	0.3	Increasing from 20 to 50
3-9	0.3	Increasing from 50 to 80
9-10.5	0.3	80
10.5-10.6	0.3	Decreasing from 80 to 20
10.6-13.5	0.3	20

Table S2. Mass spectrum parameters

Parameter type	Value or category
Ionization mode	ESI positive and negative ion mode
Scan type	Multiple reaction monitoring (MRM)
Curtain gas	15 psi
Spray voltage	+4500 v, -4000 V
Atomizing gas pressure	65 psi
Auxiliary gas pressure	70 psi
Atomization temperature	400 ℃

Table S3. Selected reaction monitoring conditions for protonated or deprotonated plant hormones ($[M+H]^+$ or $[M-H]^-$)

Hormones	Polarity	Parent ion （m/z）	Daughter ion （m/z）	Decoupling voltage (V)	Collision energy (V)
GA3	-	345.2	143.0/239.2*	-80	-30/-33
ABA	-	263.1	153.1*/204.2	-60	-14/-27
IAA	+	176.2	129.8*/102.9	65	12/42
JA	-	209.2	59.1*	-54	-16
SA	-	137	92.9*/65	-50	-20/-39
IBA	-	202	116.1*/158/184	-80	-20/-18/-19

Note: Those marked with “*” are quantitative ions.
